# Mitochondrial respiratory chain component NDUFA4: a promising therapeutic target for gastrointestinal cancer

**DOI:** 10.1186/s12935-024-03283-8

**Published:** 2024-03-05

**Authors:** Quanling Zhou, Xiaohui Li, Honglian Zhou, Juanjuan Zhao, Hailong Zhao, Lijuan Li, Ya Zhou

**Affiliations:** 1 Department of Pathophysiology, Zunyi Medical University, Zunyi, 563000 Guizhou China; 2 Department of Physics, Zunyi Medical University, Zunyi, 563000 Guizhou China; 3 Key Laboratory of Gene Detection and Therapy of Guizhou Province, Zunyi, 563000 Guizhou China

**Keywords:** Gastrointestinal cancer, NDUFA4, Colorectal cancer, Liver cancer, Gastric cancer, Esophageal cancer, Pancreatic cancer, Regulatory mechanism

## Abstract

Gastrointestinal cancer, one of the most common cancers, continues to be a major cause of mortality and morbidity globally. Accumulating evidence has shown that alterations in mitochondrial energy metabolism are involved in developing various clinical diseases. NADH dehydrogenase 1 alpha subcomplex 4 (NDUFA4), encoded by the *NDUFA4* gene located on human chromosome 7p21.3, is a component of mitochondrial respiratory chain complex IV and integral to mitochondrial energy metabolism. Recent researchers have disclosed that NDUFA4 is implicated in the pathogenesis of various diseases, including gastrointestinal cancer. Aberrant expression of NDUFA4 leads to the alteration in mitochondrial energy metabolism, thereby regulating the growth and metastasis of cancer cells, indicating that it might be a new promising target for cancer intervention. This article comprehensively reviews the structure, regulatory mechanism, and biological function of NDUFA4. Of note, the expression and roles of NDUFA4 in gastrointestinal cancer including colorectal cancer, liver cancer, gastric cancer, and so on were discussed. Finally, the existing problems of NDUFA4-based intervention on gastrointestinal cancer are discussed to provide help to strengthen the understanding of the carcinogenesis of gastrointestinal cancer, as well as the development of new strategies for clinical intervention.

## Introduction

According to the latest global cancer burden report released by the World Health Organization (IARC) in 2020, there were 19.3 million new cases of cancer worldwide, resulting in almost 10 million deaths. Gastrointestinal cancer, encompassing gastric, colorectal, hepatic, and esophageal cancers, constitutes over 40% of all cancer cases and poses a significant threat to human well-being [1]. Despite significant advancements in clinical diagnosis and treatment strategies for gastrointestinal cancers, such as the utilization of small molecule inhibitors like regorafenib and immune checkpoint inhibitors (ICIs) represented by anti-PD-1 monoclonal antibodies, there is still a need to improve clinical interventions for these cancers due to the complexity of their pathogenesis [2]. Therefore, a deeper understanding of the underlying mechanisms of gastrointestinal cancer is still essential for identifying new clinical treatment targets in the future.

Energy metabolism (EM) is crucial for cellular growth and function, encompassing intricate processes such as glycolysis and oxidative phosphorylation (OXPHOS) to generate adenosine triphosphate (ATP). Researchers have demonstrated that cancer cell survival, proliferation, invasion, metastasis, and drug resistance are energy-demanding phenomena driven by glycolysis and OXPHOS metabolic pathways [3]. Among these processes, the shift from OXPHOS to aerobic glycolysis (the Warburg effect: Warburg found that, unlike most normal tissues, cancer cells tend to “ferment” glucose to lactic acid even when there is enough oxygen to support mitochondrial oxidative phosphorylation) is critical in promoting the biological behaviors of cancer cells [4]. As a result, the study of cancer mitochondrial energy metabolism has become a hot topic in cancer research. NADH dehydrogenase 1 alpha sub-complex 4 (NDUFA4), a member of the NDUFA family, is an essential component of the mitochondrial respiratory chain and is responsible for redox processes and ATP production. Recent studies have demonstrated that aberrant expression of NDUFA4 leads to the alteration in mitochondrial energy metabolism, thereby regulating the growth and metastasis of various types of cancer cells including gastrointestinal cancer cells [5]. These findings suggest that NDUFA4 might be an important new target for clinical interventions for gastrointestinal cancer.

## The structure, function, and regulatory mechanisms of NDUFA4

### The structure and expression of NDUFA4

**Fig. 1:**
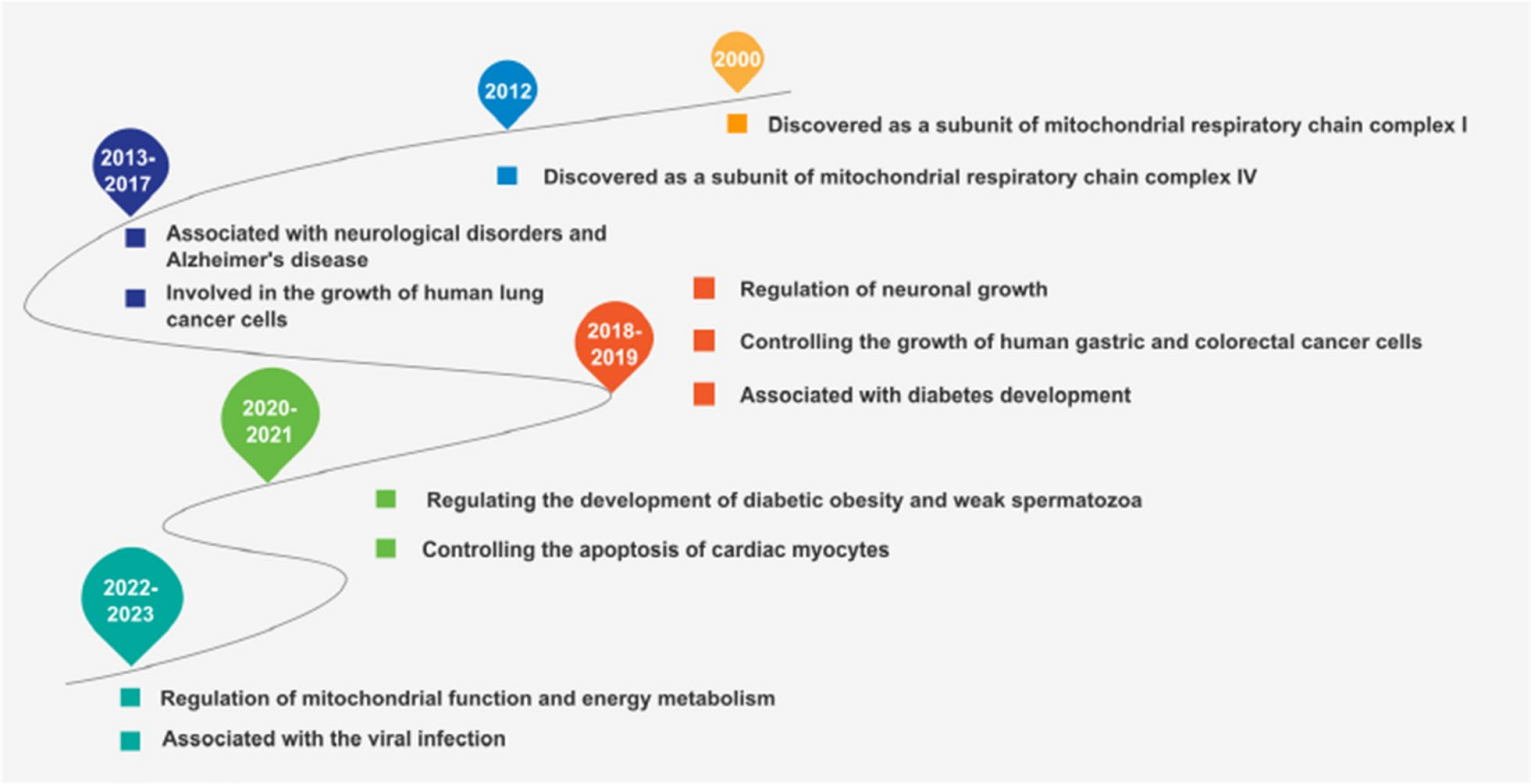
Timeline of the research progression of NDUFA4

**Fig. 2:**
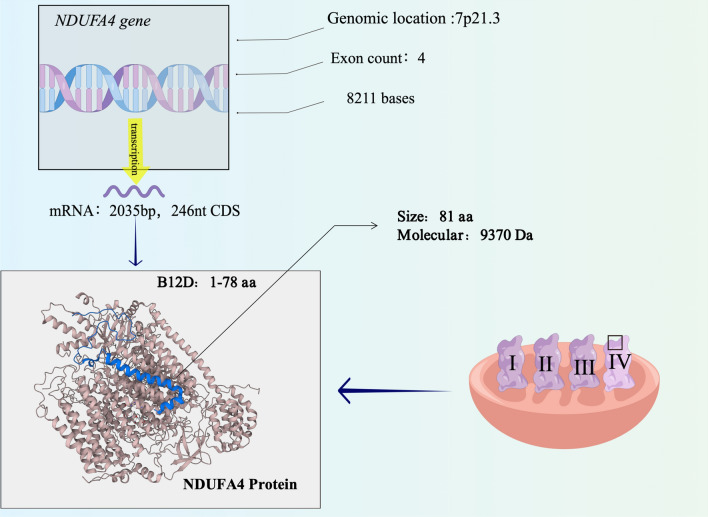
The structure and regulatory mechanisms of NDUFA4 (By Figdraw)

NDUFA4 is a crucial protein encoded by the *NDUFA4* gene located on human chromosome 7p21.3, which plays an important role in the mitochondrial respiratory chain. The *NDUFA4* gene is 8211 base pairs long, with a molecular weight of approximately 9 kb, and contains four exons. The mRNA of *NDUFA4* is 2,035 nt in length, including a coding DNA sequence (CDS) of 246 nt. The NDUFA4 protein comprises 81 amino acids with a molecular weight of 9370 Da and contains the domain of NADH-ubiquinone reductase complex 1MLRQ subunit (B12D). Although earlier studies suggested that NDUFA4 is a subunit of complex I [6], recent studies have confirmed that it is a subunit of COX and can interact with COX itself. This indicates that NDUFA4 is an essential component in the respiratory chain [7–10]. (Figs. 1, 2).

NDUFA4 is ubiquitously expressed in the body tissues and organs, including the brain, heart, digestive, epidermis, nervous, respiratory, and skeletal muscle tissues. However, its expression level varies significantly depending on the tissue types [11]. For instance, Garbian et al. found that NDUFA4 was significantly highly expressed in liver and brain tissues [6]. In addition, the expression of NDUFA4 was also higher in the parathyroid gland, gastric, duodenum, and myocardium. However, NDUFA4 expression is lower in cervical and rectal tissues. These findings suggest that the expression pattern of NDUFA4 is closely related to the status of the energic metabolism of tissues and organs.

The expression of NDUFA4 is regulated by multiple mechanisms, including the post-translational level and protein modification. Several miRNAs have been identified that regulate the expression of NDUFA4. For example, miR-210-3p targets the 3’UTR of NDUFA4 mRNA in cardiomyocytes, resulting in impaired mitochondrial function and thereby promoting cardiomyocyte apoptosis [12]. Posttranslational modifications also play a crucial role in regulating NDUFA4 expression. Studies have shown that the NDUFA4 protein has multiple ubiquitination sites, including Lys55, Lys63, Lys73, and Lys75, which affect its stability (from www.nextprot.org [NX_O00483-1]). In addition, bioinformatics analysis shows that there are several putative transcription factor-binding sites on the *NDUFA4 gene* promoter, including Foxj2, GR, HNF-4a1, HNF-4a2, and LCR-F1, indicating that transcriptional regulation also might be involved in the regulatory mechanism of NDUFA4 expression. Therefore, further studies are needed to determine the exact role and mechanism of NDUFA4 regulation in distinct tissues and organs.

### The biological function of NDUFA4

NDUFA4 is an essential enzyme located at the end of the mitochondrial electron transport chain, and responsible for NADH dehydrogenase and oxidoreductase activities by transferring electrons from NADH to the respiratory chain, thus driving OXPHOS and participating in respiratory electron transport, chemiosmotic coupling to ATP, and the uncoupling of proteins to generate heat. Meanwhile, NDUFA4 can also be involved in the cellular stress response [13–15]. Abnormal expression of NDUFA4 has been linked to a range of clinical disorders (Table 1), including mitochondrial complex IV deficiency nuclear type 21 and Leigh syndrome, which are autosomal recessive disorders.

**Table 1:** Relationship between NDUFA4 expression and development of some diseases

Classification	Disease	Expression of NDUFA4	Regulation result	Conclusion	References
Cancers	Colorectal cancer (CRC)	Overexpression	Promotes the in vitro growth of human CRC cancer cells with altered mitochondrial energy metabolism	NDUFA4 assumes a pivotal role in the development of human CRC by regulating the OXPHOS and glycolysis, as well as other molecules, indicating its complex role in human CRC	[29–33]
		Low expression	Inhibits the in vitro growth of human CRC cancer cells		
	Gastric cancer (GC)	Overexpression	Promotes GC cell growth	NDUFA4 assumes a pivotal role in the development of GC and participates in the regulation of other molecules, reflecting its potential application value in the prognosis and treatment of GC	[11, 34–36]
		Low expression	Inhibits cancer growth		
	Esophageal squamous cell carcinoma (ESCC)	Overexpression	Inhibit the proliferation and invasion of ESCC and alters the cell cycle distribution	NDUFA4 is closely related to ESCC through regulation of the growth and metastasis of human ESCC cells	[37]
		Low expression	–		
	Pancreatic adenocarcinoma (PAAD)	Overexpression	Improve the proliferation of human pancreatic cancer cells and promote the growth of cancer cells in vivo	NDUFA4 is upregulated in pancreatic cancer tissues and negatively correlated with patient survival	[38]
		Low expression	Induces the opposite effect		
	Lung cancer (LCA)	Overexpression	Increases the growth and metastasis of human lung cancer cells	NDUFA4 promotes the growth and metastasis of human lung cancer cells and leads to alterations in Akt and Erk pathway signaling. The optimized TTF-1 promoter can more effectively manipulate miR-7 to affect the growths of human non-small cell lung cancer (NSCLC) cells via inhibiting NDUFA4 expression	[25, 39]
		Low expression	Induces the reduction in human lung cancer cell growth and metastasis due to miR-7 expression		
	Breast cancer (BC)	Overexpression	Enhances OXPHOS pathway and increases ATP consumption	Metabolic conversion is a key alteration in the ecological niche of breast cancer cells prior to preparation for metastasis	[40]
	Renal cell carcinoma (RCC)	Overexpression	Higher expression in distal tubules	NDUFA4 is differentially expressed in renal cell carcinoma and is associated with cancer-specific survival	[41]
		Low expression	NDUFA4 mRNA and protein downregulation		
	Head and neck paraganglioma (HNPGL)	Overexpression	Promotes the assembly of mitochondrial respiratory chain complexes, increases ATP production, and elevates cancer cell viability	NDUFA4 promotes the progression of HNPGL	[42]
		Low expression	Impairs the assembly of mitochondrial respiratory chain complexes and decreases the production of ATP and reduced cancer cell viability		
Genetic diseases	Diabetic obesity	Low expression	Impairs glucose uptake and mitochondrial complex IV activity	Adipose tissue macrophage-derived miR-210 regulates glucose uptake and mitochondrial complex IV activity by targeting NDUFA4 expression to promote the development of obesity in diabetic mice	[43]
		Overexpression	Opposite of above		
	Diet-induced diabetes	Low expression	Enhances oxidative stress	NDUFA4 mutation is directly associated with mitochondrial dysfunction, which, together with SDF2L1 deletion expression and diabetic diet, leads to enhanced oxidative stress, preventing mitochondrial ATP production and thereby impairing the ability of the pancreas to secrete insulin and leading to the development of diabetes	[44–46]
Heterogeneous diseases	Alzheimer's disease (AD)	Expression	NDUFA4 is associated with mitochondrial dysfunction in the pathogenesis of AD	Complex IV of the mitochondrial electron transport chain (cytochrome c oxidase, COX) is particularly vulnerable in AD. mRNA levels of NDUFA4 correlate significantly with Aβ plaque load in the hippocampus of AD mice	[47–50]
	Azoospermia (AS)	Overexpression	Reduces the level of DJ-1 (a protein highly associated with male sterility)	DJ-1 deficiency in testicular tissue may be closely related to the localization of NDUFS3 and the level of NDUFA4, leading to abnormalities in mitochondrial energy metabolism and multiple other metabolic pathways	[51]
	Amyotrophic lateral sclerosis (ALS)	NDUFA4 binds to receptor expression enhancing protein (REEP1)	Maintains mitochondrial complex IV function	Motor function is preserved in SOD1 G93A mice	[52]
Infectious diseases	Viral infections (Zika virus ZIKV, dengue virus, SARS-CoV-2)	Low expression	Leads mitochondrial stress, which leads to mtDNA leakage and the upregulation of type I interferon signaling. Isogenic human induced pluripotent stem cell (hiPSC) lines carrying nonrisk alleles of single nucleotide polymorphisms (SNPs) or cis-regulatory region deletions are less sensitive to viral infection	NDUFA4 is identified as a previously unknown susceptibility locus for viral infection	[53]
Body functions	Neurons	Overexpression	Inhibits miR-145a-5p expression can promote neuronal proliferation and inhibit neuronal apoptosis in vitro	NDUFA4 promotes the proliferation and inhibits the apoptosis of neurons by inhibiting miR-145a-5p	[28]
		Low expression	Enhances Mir-145a-5p expression, thereby inhibiting the proliferation of neurons and promoting their apoptosis		
	Heart function	Expression	Sustains mitochondrial function	CLOCK regulates adaptive stress responses critical for cell survival by transcriptionally orchestrating mitochondrial quality control mechanisms in cardiomyocytes	[54]

NDUFA4 has also been identified as a candidate gene for the sex-specific inheritance of diabetes in rodent models [16–19]. Besides, *NDUFA4 gene* silencing is associated with the response to abatacept (AB) in patients with rheumatoid arthritis (RA) [20]. Similarly, *the NDUFA4 gene* has also been identified as a central gene associated with heterocyclic amine-induced cytotoxicity in peripheral blood monocytes [21]. High expression of NDUFA4 is significantly associated with poorer overall survival (OS) in patients with bacterial sepsis [22]. Liao C et al. showed that Dandy-Walker malformation might be caused by insufficient haploid or overexpression of the *NDUFA4 gene* [23, 24]. In line with these findings, NDUFA4 dysfunction in related tissues has also been implicated in the musculoskeletal system and hearing impairments.

Moreover, recent research has shown that NDUFA4 can affect cellular energy metabolism by regulating the PI3K/Akt signaling pathways. The PI3K/Akt pathway plays a role in glycolysis by regulating the hypoxia-inducible factor (HIF)-1α target genes ENO1 and LDHA, suggesting that NDUFA4 may not only directly participate in mitochondrial redox processes but also impact cellular energy metabolism by modulating signaling pathway transduction including Akt and Erk pathway [25–27].

Additionally, Fu F et al. demonstrated that NDUFA4 can promote neuron proliferation and inhibit apoptosis by suppressing the expression of miR-145a-5p [28]. Of note, recent studies have further shown that NDUFA4 is aberrantly expressed in various cancers, including gastrointestinal cancer, and is involved in cancer cell growth, metastasis, and drug resistance. These findings suggest that NDUFA4 might be a new target for clinical interventions for cancers.

## NDUFA4 and gastrointestinal cancer

### Colorectal cancer

Colorectal cancer (CRC) ranks among the most common gastrointestinal cancers with high morbidity and mortality rates [1]. Abnormal expression of NDUFA4 has been found in human CRC, where NDUFA4 has been shown to regulate the growth and metastasis of cancer cells, indicating that it is a potential new target for intervention. For example, Shiming Liu et al. found that NDUFA4 was highly expressed in human CRC cancer tissues, and its overexpression promoted the in vitro growth of human CRC cancer cells with altered mitochondrial energy metabolism [29]. Mechanistic aspects: NDUFA4 collaborates with leucine-rich pentatricopeptide repeat containing (LRPPRC) to regulate the transmission of signaling pathways such as the Akt and Erk pathways, ultimately leading to mitochondrial ATP changes. Meanwhile, NDUFA4 was found to have a positive correlation with the expression of LRPPRC in human CRC tissues. Furthermore, the overexpression of NDUFA4 has been demonstrated to facilitate epithelial-mesenchymal transition (EMT) in human CRC cells [30]. Besides, Yun L et al.found that NDUFA4L2 was significantly enriched in the mitochondria under hypoxic conditions and was associated with cancer progression and poor prognosis in human CRC patients [31] (In other diseases, during inflammation, a cytokine modulator of cytochrome C oxidase (MOCCI), a para homolog of NDUFA4, replaces NDUFA4 during inflammation to reduce mitochondrial membrane potential and reduce ROS production, leading to cell protection and the suppression of the immune response [55]).

Studies have further revealed that NDUFA4 is involved in the regulatory process of other molecules in human CRC cancer cells. For example, Wu et al. found that NDUFA4 may be a new proteolytic substrate of the mitochondrial protease OMA1. Under hypoxic conditions, OMA1 increases the production of mitochondrial reactive oxygen species (mtROS) production, promotes glycolysis, and inhibits mitochondrial OXPHOS by promoting the degradation of NDUFA4 in CRC cells in vivo and in vitro, which impairs the mitochondrial respiratory complex, resulting in increased lactate production and glucose uptake, and decreased ATP production [32]. Additionally, Cui et al.found that NDUFA4 was a direct target of miR-147b. Knockdown of NDUFA4 attenuated the cancer-promoting effect caused by miR-147b downregulation. Further studies have revealed that the cancer-promoting lncRNA MAFG-AS1 regulates the miR-147b/NDUFA4 axis in human CRC. NDUFA4 promotes glycolysis over OXPHOS in CRC cells by regulating glycolysis-related genes. The investigation indicates that MAFG-AS1 and NDUFA4 collectively enhance CRC cell invasion and increase lactate production, implicating these molecules in the regulatory networks governing CRC metabolic and tumorigenic characteristics [33].

In summary, NDUFA4 assumes a crucial role in the development of human CRC by modulating the Akt and Erk signaling pathways and glycolysis, as well as other molecules. However, further studies are needed to investigate whether NDUFA4 is involved in human CRC stem cell development and the drug resistance of human CRC cancer cells. Such investigations hold the promise of crucial insights for the formulation of relevant intervention strategies.

### NDUFA4 and liver cancer

Liver cancer (LC) is a leading cause of cancer-related mortality worldwide. Recent studies have demonstrated that the NDUFA4 molecule NDUFA4L2 is aberrantly expressed in hepatocellular carcinoma (HCC) and is involved in the intricate regulation of cancer cell growth and metastasis. For instance, Lai R et al. reported that NDUFA4L2 was significantly upregulated in HCC cancer tissues compared with adjacent tissues. Moreover, the upregulation of NDUFA4L2 expression was more significant under hypoxia in HCC [56]. Tello D et al. further found that NDUFA4L2 reduced mitochondrial oxygen consumption and complex I activity, thereby reducing ROS production and accumulation. This ultimately leads to decreased cancer cell apoptosis and promotes cell survival [57]. However, the knockdown of NDUFA4L2 can significantly inhibit the growth and metastasis of HCC in vivo, indicating that the high expression of NDUFA4L2 is conducive to the hypoxia tolerance of cancer cells [56] (Over 90% of clear cell renal cell carcinomas (ccRCCs) exhibit overexpression of NDUFA4L2, which facilitates ccRCC proliferation and survival. Meanwhile, NDUFA4L2 can regulate mitochondrial and lysosome functions in ccRCC [58]).

Regarding expression regulation, studies have shown that the upregulation of NDUFA4L2 in HCC cells is closely related to HIF. Under hypoxic conditions, cancer cells upregulate HIF -1ɑ expression, consequently leading to increased NDUFA4L2 expression and reduced ROS accumulation. This suggests a dependency of NDUFA4L2 expression on HIF1 in HCC cells. Inactivation of HIF1/NDUFA4L2 increased mitochondrial activity and oxygen consumption, resulting in ROS accumulation and cell apoptosis. In addition, HIF-1α steadily increases miR-210, leading to mitochondrial dysfunction of the echinotrophoblast (EVT) in early pregnancy. The *NDUFA4 gene* is a significant inhibitory gene in early pregnancy primary echinotrophoblasts transfected with miR-210 and may be related to the pathogenesis of preeclampsia [59]. These results suggest that NDUFA4 may play a pivotal role in developing HCC by regulating mitochondrial function.

Regarding clinical prognosis, studies have shown that high expression of NDUFA4L2 is closely associated with microsatellite stability and cancer encapsulation in HCC and with a poor prognosis of HCC patients, indicating its potential value in the clinical diagnosis and treatment of HCC. These findings suggest that NDUFA4 may be a potential new target for HCC clinical treatment. Therefore, further exploration of the molecular mechanism of NDUFA4 regulation of HCC is crucial for the development of relevant intervention strategies.

### NDUFA4 and gastric cancer

Gastric cancer (GC) is the fourth leading cause of cancer-related deaths worldwide, following lung and liver cancer [1]. Studies have shown that NDUFA4 is highly expressed in human GC and regulates cancer cell growth and metastasis. For example, researchers used single-cell and bulk RNA-seq, as well as tissue microarray technology, to detect and found that the expression of NDUFA4 was upregulated in cancer tissues, which was positively correlated with the poor prognosis of patients [60, 61].

Regarding related metabolism, studies have revealed that the inhibition of glycolysis inhibits GC cell proliferation and cancer growth. Xu et al. Further showed that NDUFA4 promoted glycolysis and oxidative metabolism in GC cells and enhanced GC cell growth by inhibiting ROS levels and increasing matrix metalloproteinase (MMP) expression. However, the inhibition of mitochondrial fission reversed NDUFA4-induced glycolysis, oxidative metabolism, and cancer growth [11]. Further studies found that the upregulation of NDUFA4 expression could accelerate the glycolysis process of human GC cancer cells, producing more ATP to meet the demand for cancer growth [34, 35]. These results suggest that targeted knockdown of NDUFA4 may be a potential new strategy to intervene in GC cancer cell growth.

Recent investigations have elucidated the regulatory role of NDUFA4 in the growth and metastasis of human GC cancer cells through intricate molecular interaction. For instance, Xu et al. found that NDUFA4 expression levels are increased through N6-methyladenosine (M6A) methylation, consequently fostering GC development by augmenting cell glycolysis and promoting mitochondrial fission. Notably, the depletion of NDUFA4 resulted in substantial inhibition of glucose uptake in cancer cells. This downregulation of NDUFA4 significantly attenuated both the extracellular acidification rate (ECAR) and the oxygen consumption rate (OCR) in GC cells, underscoring its pivotal role in cellular energetics. Furthermore, the reduction of NDUFA4 levels was also correlated with a significant decrease in intracellular lactate and ATP concentrations [11]. Li et al. found the role of the LNCMIF-AS1/NDUFA4/MIR-212-5P axis in the occurrence and development of gastric cancer. lncMIF-AS 1 positively regulates the expression of NDUFA4 in gastric cancer cells by attenuating the degradation or repression of the NDUFA4 mRNA induced by MIR-212-5P [36]. The upregulation of NDUFA4 significantly enhances the proliferation ability and inhibits the apoptosis rate of gastric cancer cells i*n vitro* by activating the OXPHOS pathway.

In conclusion, NDUFA4 emerges as a pivotal player in the development of GC through the regulation of mitochondrial OXPHOS and glycolysis pathways and participates in the regulation of other molecules, reflecting its potential application value in the prognosis and treatment of GC. However, the relationship between NDUFA4 expression in GC and drug resistance in human GC stem cells and GC cancer cells needs further investigation.

### NDUFA4 and other cancers

Esophageal squamous cell carcinoma (ESCC) represents the most common type of esophageal cancer (EC). Recent studies have revealed that the relative expression level of NDUFA4 in ESCC tissues is significantly lower than that in adjacent tissues. Moreover, the expression of NDUFA4 is closely related to the clinical stage, invasion depth, histological grade, and lymph node metastasis of ESCC. Some studies have further demonstrated that NDUFA4 is involved in the regulation of the growth and metastasis of human ESCC cancer cells. For instance, Y Tang et al. found that NDUFA4 was a direct target gene of miR-147b, and the expression of NDUFA4 in ESCC tissues was negatively correlated with the expression of miR-147b. The miR-147b inhibitor significantly increased the expression of NDUFA4 in ESCC EC1 and EC9706 cells, inhibiting the proliferation and invasion of ESCC and altering the cell cycle distribution [37].

Pancreatic adenocarcinoma (PAAD) is the third leading cause of cancer-related death, with a 5-year relative survival rate of 6%. Recent studies have indicated that NDUFA4 is also upregulated in PAAD tissues, and its expression level demonstrates a negative correlation with patient survival. Zhang et al. further found that NDUFA4 knockdown could induce G1 phase arrest to reduce the proliferation of human PAAD cells [38]. Mechanistically, NDUFA4 knockdown reduced the oxygen consumption rate, cellular ATP level, mitochondrial complex IV activity, and related protein levels, thereby inhibiting OXPHOS. Conversely, NDUFA4 overexpression led to the opposite effects. These results suggest that NDUFA4 promotes pancreatic cancer proliferation by enhancing OXPHOS.

Verrucous epidermal nevus (VEN) is a benign skin cancer that appears at birth or early childhood. NDUFA4 is up-regulated in the lesions of children with VEN, which may be related to the pathogenesis of VEN [62]. In addition, Head and neck paragangliomas (HNPGLs) are rare cancers in which the expression of NDUFA4 shows a significant increase in expression [63], indicating that NDUFA4 also might be involved in the development of HNPGLs.

**Fig. 3:**
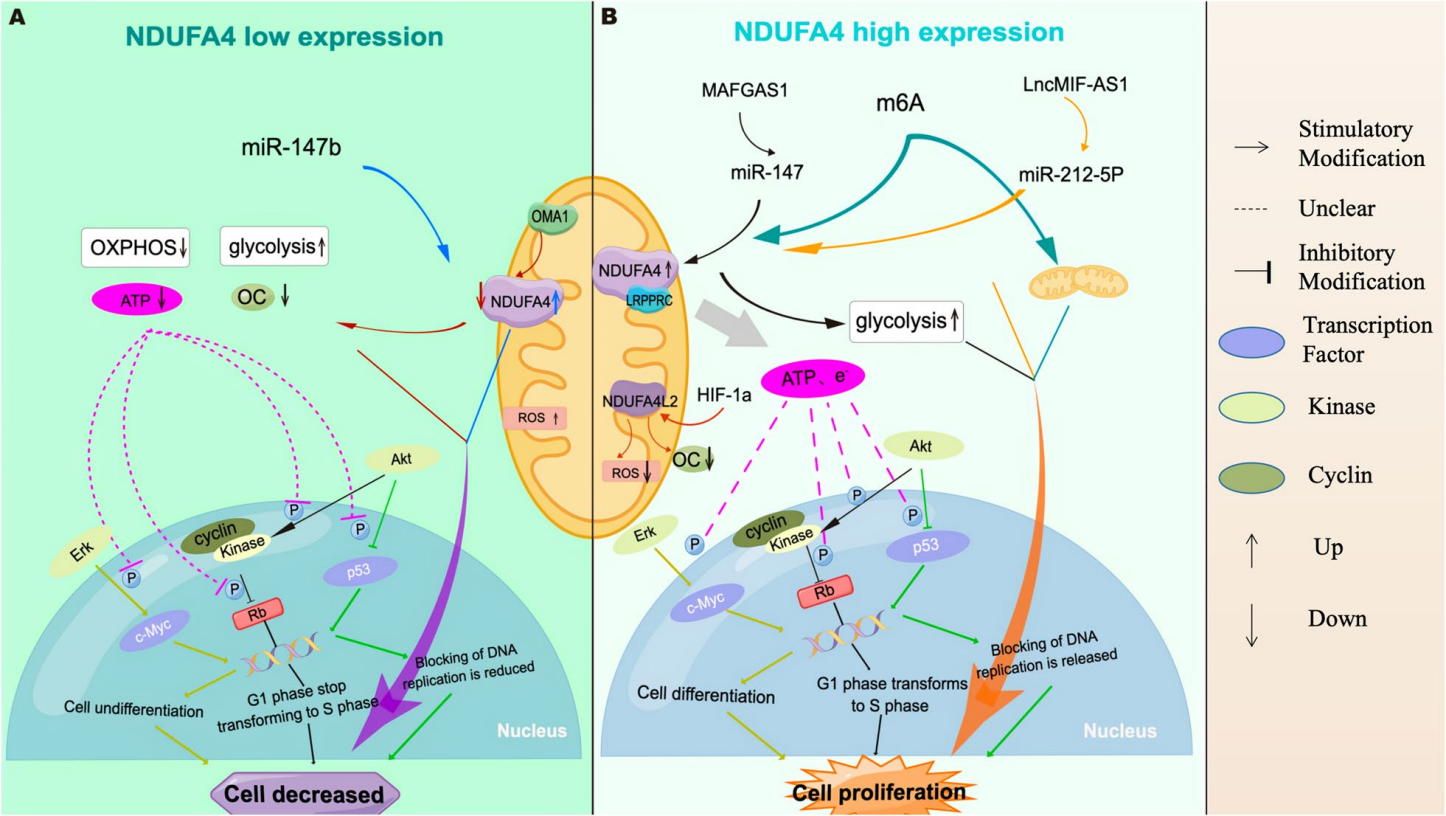
Schematic map of the mechanisms by which NDUFA4 regulates the proliferation of gastrointestinal cancer cells. **A** Low NDUFA4 or downregulated expression leads to decreased cell number. **B** High expression of NDUFA4 or its regulated expression leads to cell proliferation (By Figdraw)

In summary, NDUFA4 plays a significant role in the occurrence and development of esophageal cancer, pancreatic cancer, and other metabolic system cancers and may serve as a new target for related cancer interventions. However, the mechanisms underlying NDUFA4 regulation in these types of cancers, particularly its aberrant expression, require further elucidation (Fig. 3).

## Summary and prospects

NDUFA4 is an essential component of the respiratory chain and maintains mitochondrial function. Aberrant expression of NDUFA4 is associated with the progression of various diseases. In gastrointestinal cancer, accumulating evidence has shown that NDUFA4 and its accessory protein NDUFA4L2 regulate OXPHOS and glycolysis, thereby affecting the growth and metastasis of cancer cells, indicating it might be a promising new target for cancer interventions. Although the related molecular mechanism of NDUFA4 in the development of gastrointestinal cancer has been investigated, many key questions remain unanswered.

First, further investigations are needed to determine the expression pattern of NDUFA4 in different gastrointestinal cancers and its transcriptional regulation. Although multiple potential transcription factor-binding sites are present on the NDUFA4 promoter, the transcriptional regulatory mechanisms underlying its aberrant expression in gastrointestinal cancers are still largely unknown. Recent studies have shown that mRNA modifications (e.g., M6A modifications) can upregulate NDUFA4 expression [11]. Meanwhile, microRNA-7 (miR-7) can downregulate NDUFA4 expression at the transcriptional level [25]. In addition, further studies showed that lncMIF-AS 1 positively regulated NDUFA4 expression by attenuating MIR-212-5P-induced NDUFA4 mRNA degradation or repression [36]. However, further investigations on the regulatory mechanism of NDUFA4 expression will provide insight into the mechanism of NDFUA4 involvement in tumorigenesis and will also enlarge our understanding of the process of tumorigenesis of gastrointestinal cancers.

Second, the role of NDUFA4 in regulating the occurrence and development of gastrointestinal cancers needs to be fully elucidated. Mitochondrial energy metabolism involves mitochondrial dynamics, signal transduction processes, and intracellular metabolic processes such as glucose, lipid, and protein metabolism. As a crucial component of mitochondrial energy metabolism, the effect of NDUFA4 on mitochondrial dynamics and related signal transmission remains largely unknown, especially considering the variations in sugar, fat, and protein metabolism involved in mitochondrial energy metabolism across different cancers. Therefore, utilizing recently developed metabolomic techniques, scRNA sequencing, and other technologies to systematically study the precise mechanism of NDUFA4 in regulating various types of gastrointestinal cancers will provide insights into the role of energy metabolism molecules, represented by NDUFA4, in the occurrence and development of malignant s.

**Fig. 4:**
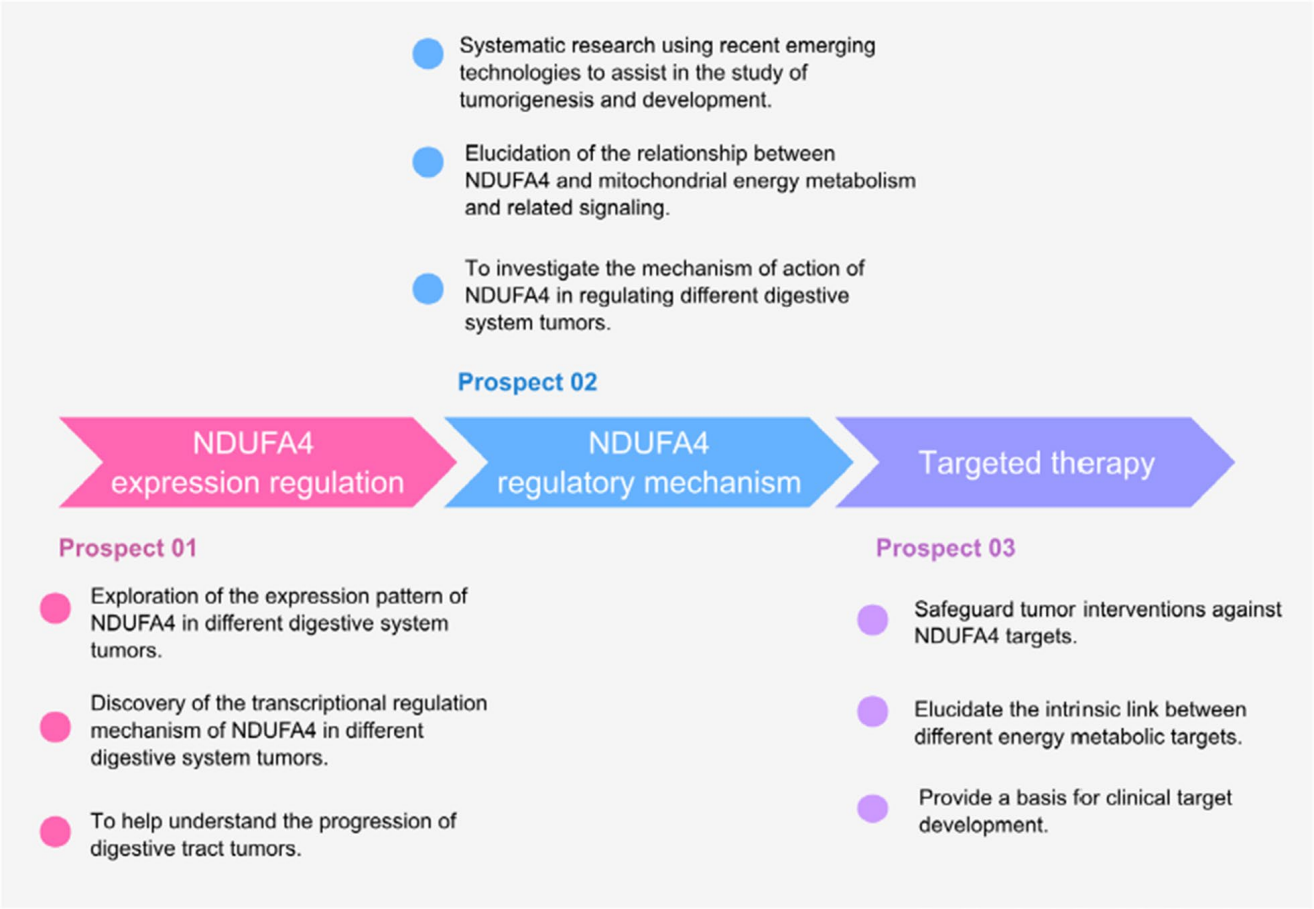
Prospects for future research works on NDUFA4

Finally, the exploration of the possible application of NDUFA4-based therapy is essential. Various targets in tumor energy metabolism, including PARP and Srit, have become new focuses for clinical disease interventions, particularly in the context of malignant cancers (Table 2). However, given the importance of NDUFA4 in the development and functional regulation of vital tissues and cells, caution is required when developing new strategies for cancer interventions targeting NDUFA4, emphasizing precision and effectiveness. The internal relationships among different energy metabolism targets also need to be elucidated to provide a crucial foundation for the final development of targeted interventions (Fig. 4).

**Table 2:** Possible therapeutic targets related to energy metabolism

Potential therapeutic targets	References
NDUFA4	[25, 32, 33, 38, 42, 43]
Response gene to complement 32 protein (RGCC)	[64]
Cepharanthine (CEP)	[65]
The muscle isoform of pyruvate kinase PKM2	[66]
Homocysteine-inducible ER protein with ubiquitin-like domain 1 (HERPUD1)	[67]
Dibenzyl diselenide (DBDS) and statins	[68]
Histone methyltransferase, suppressor of variegation 3–9 homolog 1 (SUV39H1)	[69]
A part of the cytoplasmic protein complex Lsm1-7-Pat1 (LSM1)	[70]
Euphorbia factor L1 (EFL1)	[71]

In conclusion, NDUFA4 and its related molecules play crucial regulatory roles in the occurrence and development of gastrointestinal cancers. Advances in genomic, RNA omic, and metabolomic techniques promise to deepen understanding of the expression regulation mechanism of NDUFA4 and its complex-related molecules in gastrointestinal cancers, as well as their relationship with clinical diseases, will be further enhanced. This will undoubtedly provide important insights into the biological functions of NDUFA family members, including NDUFA4, and is helpful for the development of new strategies for preventing and treating related clinical diseases.

## Data Availability

Not applicable.

## Abbreviations

NDUFA4: NADH dehydrogenase 1 alpha subcomplex 4
EM: Energy metabolism
ICIs: Immune checkpoint inhibitors
B12D: NADH-ubiquinone reductase complex 1MLRQ subunit
KW-0812: Transmembrane β-chain domain
COX: Cytochrome-c oxidase
CRC: Colorectal cancer
LRPPRC: Leucine-rich pentatricopeptide repeat containing
EMT: Epithelial-mesenchymal transition
NDUFA4L2: NDUFA4 mitochondrial complex 2
mtROS: Mitochondrial reactive oxygen species
OXPHOS: Oxidative phosphorylation
LC: Liver cancer
HCC: Hepatocellular carcinoma
GC: Gastric cancer
MMP: Matrix metalloproteinase
M6A: N6-methyladenosine
ESCC: Esophageal squamous cell carcinoma
PAAD: Pancreatic adenocarcinoma
VEN: Verrucous epidermal naevi
HNPGLs: Head and neck paragangliomas
AB: Abatacept
RA: Rheumatoid arthritis
OS: Overall survival
MOCCI: The modulator of cytochrome C oxidase
ccRCC: Clear cell renal cell carcinoma
EVT: Early pregnancy primary echinotrophoblast
LCA: Lung cancer
BC: Breast cancer
RCC: Renal cell carcinoma
AD: Alzheimer’s disease
AS: Azoospermia
ALS: Amyotrophic lateral sclerosis

## References

[1] Sung H (2021). Global cancer statistics 2020: GLOBOCAN estimates of incidence and mortality worldwide for 36 cancers in 185 countries. CA Cancer J Clin.

[2] Pavlakis N (2016). Regorafenib for the treatment of advanced gastric cancer (INTEGRATE): a multinational placebo-controlled phase II trial. J Clin Oncol.

[3] Liu W (2019). Weipiling ameliorates gastric precancerous lesions in Atp4a(–/–) mice. BMC Complement Altern Med.

[4] Zhao X (2018). Shikonin inhibits tumor growth in mice by suppressing pyruvate kinase M2-mediated aerobic glycolysis. Sci Rep.

[5] Clayton SA (2021). Inflammation causes remodeling of mitochondrial cytochrome c oxidase mitochondrial cytochrome c oxidase remodeling mediated by the bifunctional gene C15orf48. Sci Adv.

[6] Garbian Y (2010). Gene expression patterns of oxidative phosphorylation complex I subunits are organized in clusters. PLoS ONE.

[7] Kadenbach B, Hüttemann M (2015). The subunit composition and function of mammalian cytochrome c oxidase. Mitochondrion.

[8] Hayashi T (2015). Higd1a is a positive regulator of cytochrome c oxidase. Proc Natl Acad Sci U S A.

[9] Vögtle FN (2017). Landscape of submitochondrial protein distribution. Nat Commun.

[10] Zong S (2018). Structure of the intact 14-subunit human cytochrome c oxidase. Cell Res.

[11] Xu W (2022). m6A RNA methylation-mediated NDUFA4 promotes cell proliferation and metabolism in gastric cancer. Cell Death Dis.

[12] Chen D, Hou Y, Cai X (2021). MiR-210-3p enhances cardiomyocyte apoptosis and mitochondrial dysfunction by targeting the NDUFA4 gene in sepsis-induced myocardial dysfunction. Int Heart J.

[13] Balsa E (2012). NDUFA4 is a subunit of complex IV of the mammalian electron transport chain. Cell Metab.

[14] Hayashi T (2009). DJ-1 binds to mitochondrial complex I and maintains its activity. Biochem Biophys Res Commun.

[15] Vernet R (2022). Identification of novel genes influencing eosinophil-specific protein levels in asthma families. J Allergy Clin Immunol.

[16] Pitceathly RDS, Taanman JW (2018). NDUFA4 (renamed COXFA4) Is a cytochrome-c oxidase subunit. Trends Endocrinol Metab.

[17] Fu F (2018). NDUFA4 enhances neuron growth by triggering growth factors and inhibiting neuron apoptosis through Bcl-2 and cytochrome C mediated signaling pathway. Am J Transl Res.

[18] Kadenbach B (2017). Regulation of mammalian 13-subunit cytochrome c oxidase and binding of other proteins: role of NDUFA4. Trends Endocrinol Metab.

[19] Barkalifa R, Yagil Y, Yagil C (2010). Sex-specific genetic dissection of diabetes in a rodent model identifies Ica1 and Ndufa4 as major candidate genes. Physiol Genomics.

[20] Derambure C (2017). Pre-silencing of genes involved in the electron transport chain (ETC) pathway is associated with responsiveness to abatacept in rheumatoid arthritis. Arthritis Res Ther.

[21] Li X (2021). Bioinformatic analysis identified hub genes associated with heterocyclic amines induced cytotoxicity of peripheral blood mononuclear cells. Genes (Basel).

[22] Wu H (2021). Identification of key genes associated with sepsis patients infected by *Staphylococcus aureus* through weighted gene co-expression network analysis. Am J Transl Res.

[23] Liao C (2012). Prenatal diagnosis and molecular characterization of a novel locus for Dandy–Walker malformation on chromosome 7p21.3. Eur J Med Genet.

[24] Liao C (2012). Dandy–Walker syndrome and microdeletions on chromosome 7. Zhonghua Yi Xue Yi Chuan Xue Za Zhi.

[25] Lei L (2017). Targeted expression of miR-7 operated by TTF-1 promoter inhibited the growth of human lung cancer through the NDUFA4 pathway. Mol Ther Nucleic Acids.

[26] Pan T (2022). Immune effects of PI3K/Akt/HIF-1α-regulated glycolysis in polymorphonuclear neutrophils during sepsis. Crit Care.

[27] Reece KM (2014). Epidithiodiketopiperazines (ETPs) exhibit in vitro antiangiogenic and in vivo antitumor activity by disrupting the HIF-1α/p300 complex in a preclinical model of prostate cancer. Mol Cancer.

[28] Fu F (2023). Ndufa4 regulates the proliferation and apoptosis of neurons via miR-145a-5p/Homer1/Ccnd2. Mol Neurobiol.

[29] Liu S. The role of NDUFA4 in the proliferation of human colorectal carcinoma cells and its probable mechanism. Zunyi Medical University, 2020 (**in Chinese**)

[30] Liu S, Tao D, Lei L (2019). Role of type I coenzyme dehydrogenase 1α subcomplex 4 overexpression on epithelial-mesenchymal transition in human colorectal cancer cells. J Med Postgrad.

[31] Lv Y (2017). Overexpression of NDUFA4L2 is associated with poor prognosis in patients with colorectal cancer. ANZ J Surg.

[32] Wu Z (2021). OMA1 reprograms metabolism under hypoxia to promote colorectal cancer development. EMBO Rep.

[33] Cui S (2018). LncRNA MAFG-AS1 promotes the progression of colorectal cancer by sponging miR-147b and activation of NDUFA4. Biochem Biophys Res Commun.

[34] Gogvadze V, Orrenius S, Zhivotovsky B (2008). Mitochondria in cancer cells: what is so special about them?. Trends Cell Biol.

[35] Frezza C, Gottlieb E (2009). Mitochondria in cancer: not just innocent bystanders. Semin Cancer Biol.

[36] Li L (2018). Long non-coding RNA MIF-AS1 promotes gastric cancer cell proliferation and reduces apoptosis to upregulate NDUFA4. Cancer Sci.

[37] Tang Y, Li Z, Shi ZX (2018). Mechanisms of the suppression of proliferation and invasion ability mediated by microRNA-147b in esophageal squamous cell carcinoma. Zhonghua Yi Xue Za Zhi.

[38] Zhang Y (2022). NDUFA4 promotes cell proliferation by enhancing oxidative phosphorylation in pancreatic adenocarcinoma. J Bioenerg Biomembr.

[39] Chen S (2022). Optimized thyroid transcription factor-1 core promoter-driven microRNA-7 expression effectively inhibits the growth of human non-small-cell lung cancer cells. J Zhejiang Univ Sci B.

[40] Li H (2017). Reference component analysis of single-cell transcriptomes elucidates cellular heterogeneity in human colorectal tumors. Nat Genet.

[41] Müller FE (2015). NDUFA4 expression in clear cell renal cell carcinoma is predictive for cancer-specific survival. Am J Cancer Res.

[42] Wang Z (2023). NDUFA4 promotes the progression of head and neck paraganglioma by inhibiting ferroptosis. Biochem Cell Biol.

[43] Tian F (2020). miR-210 in exosomes derived from macrophages under high glucose promotes mouse diabetic obesity pathogenesis by suppressing NDUFA4 expression. J Diabetes Res.

[44] Yagil C (2023). Dysregulated UPR and ER stress related to a mutation in the Sdf2l1 gene are involved in the pathophysiology of diet-induced diabetes in the cohen diabetic rat. Int J Mol Sci.

[45] Yagil C, Varadi-Levi R, Yagil Y (2018). A novel mutation in the NADH dehydrogenase (ubiquinone) 1 alpha subcomplex 4 (Ndufa4) gene links mitochondrial dysfunction to the development of diabetes in a rodent model. Dis Model Mech.

[46] Yagil Y (2016). Three interacting genomic loci incorporating two novel mutations underlie the evolution of diet-induced diabetes. Mol Med.

[47] Bi R (2018). Genetic association of the cytochrome c oxidase-related genes with Alzheimer's disease in Han Chinese. Neuropsychopharmacology.

[48] Tsai SQ (2015). GUIDE-seq enables genome-wide profiling of off-target cleavage by CRISPR-Cas nucleases. Nat Biotechnol.

[49] Shim JS (2017). Global analysis of ginsenoside Rg1 protective effects in β-amyloid-treated neuronal cells. J Ginseng Res.

[50] Adav SS, Park JE, Sze SK (2019). Quantitative profiling brain proteomes revealed mitochondrial dysfunction in Alzheimer's disease. Mol Brain.

[51] Sun Y (2020). DJ-1 deficiency causes metabolic abnormality in ornidazole-induced asthenozoospermia. Reproduction.

[52] Qin S (2022). REEP1 preserves motor function in SOD1(G93A) mice by improving mitochondrial function via interaction with NDUFA4. Neurosci Bull.

[53] Han Y (2022). A human iPSC-array-based GWAS identifies a virus susceptibility locus in the NDUFA4 gene and functional variants. Cell Stem Cell.

[54] Rabinovich-Nikitin I (2021). Mitochondrial autophagy and cell survival is regulated by the circadian clock gene in cardiac myocytes during ischemic stress. Autophagy.

[55] Lee CQE (2021). Coding and non-coding roles of MOCCI (C15ORF48) coordinate to regulate host inflammation and immunity. Nat Commun.

[56] Lai RK (2016). NDUFA4L2 fine-tunes oxidative stress in hepatocellular carcinoma. Clin Cancer Res.

[57] Tello D (2011). Induction of the mitochondrial NDUFA4L2 protein by HIF-1α decreases oxygen consumption by inhibiting complex I activity. Cell Metab.

[58] Kubala JM (2023). NDUFA4L2 reduces mitochondrial respiration resulting in defective lysosomal trafficking in clear cell renal cell carcinoma. Cancer Biol Ther.

[59] Anton L (2019). HIF-1α stabilization increases miR-210 eliciting first trimester extravillous trophoblast mitochondrial dysfunction. Front Physiol.

[60] Zhang P (2019). Dissecting the single-cell transcriptome network underlying gastric premalignant lesions and early gastric cancer. Cell Rep.

[61] Cheng L (2012). Global gene expression and functional network analysis of gastric cancer identify extended pathway maps and GPRC5A as a potential biomarker. Cancer Lett.

[62] Yuan T (2021). Differentially expressed proteins identified by TMT proteomics analysis in children with verrucous epidermal naevi. J Eur Acad Dermatol Venereol.

[63] Wang Z (2021). High throughput proteomic and metabolic profiling identified target correction of metabolic abnormalities as a novel therapeutic approach in head and neck paraganglioma. Transl Oncol.

[64] Cheng S (2023). RGCC-mediated PLK1 activity drives breast cancer lung metastasis by phosphorylating AMPKα2 to activate oxidative phosphorylation and fatty acid oxidation. J Exp Clin Cancer Res.

[65] Lu YY (2023). Cepharanthine, a regulator of keap1-Nrf2, inhibits gastric cancer growth through oxidative stress and energy metabolism pathway. Cell Death Discov.

[66] Huang PC (2023). Pyruvate kinase differentially alters metabolic signatures during head and neck carcinogenesis. Int J Mol Sci.

[67] Paredes F (2023). HERPUD1 governs tumor cell mitochondrial function via inositol 1,4,5-trisphosphate receptor-mediated calcium signaling. Free Radic Biol Med.

[68] Noè R (2023). Organic selenium induces ferroptosis in pancreatic cancer cells. Redox Biol.

[69] Zhang Y (2023). SUV39H1 is a novel biomarker targeting oxidative phosphorylation in hepatitis B virus-associated hepatocellular carcinoma. BMC Cancer.

[70] Tzeng YT (2023). The role of LSM1 in breast cancer: shaping metabolism and tumor-associated macrophage infiltration. Pharmacol Res.

[71] Chen X (2023). Euphorbia factor L1 inhibited transport channel and energy metabolism in human colon adenocarcinoma cell line Caco-2. Biomed Pharmacother.

